# Perfusate hemoglobin during normothermic liver machine perfusion as biomarker of early allograft dysfunction: A pilot study

**DOI:** 10.1111/aor.14862

**Published:** 2024-09-18

**Authors:** Akinori Maeda, Graham Starkey, Sofia Spano, Anis Chaba, Glenn Eastwood, Osamu Yoshino, Marcos Vinicius Perini, Michael Fink, Rinaldo Bellomo, Robert Jones

**Affiliations:** ^1^ Department of Intensive Care Austin Hospital Melbourne Victoria Australia; ^2^ Department of Emergency and Critical Care Medicine The University of Tokyo Tokyo Japan; ^3^ Victorian Liver Transplant Unit Austin Hospital Melbourne Victoria Australia; ^4^ Department of Surgery, Austin Hospital The University of Melbourne Melbourne Victoria Australia; ^5^ Australian Centre for Transplantation Excellence and Research Austin Hosptial Melbourne Victoria Australia; ^6^ Australian and New Zealand Intensive Care Research Centre Monash University Melbourne Victoria Australia; ^7^ Data Analytics Research and Evaluation Centre Austin Hospital Melbourne Victoria Australia

**Keywords:** early allograft dysfunction, hemoglobin, ischemia reperfusion injury, liver transplantation, normothermic liver perfusion

## Abstract

**Background:**

Normothermic machine perfusion (NMP) aims to reduce ischemia–reperfusion injury in donor livers and its clinical manifestation, early allograft dysfunction (EAD) by maintaining perfusion and oxygenation. However, there is limited data on which NMP perfusate biomarkers might be associated with such EAD and the role of perfusate hemoglobin has not been assessed.

**Methods:**

We performed a pilot retrospective analysis of adult donor livers undergoing NMP between 2020 and 2022 at our center. NMP was commenced at the recipient hospital after initial static cold storage. All NMP circuits were primed in the same manner according to the manufacturer's instructions. Livers were stratified by initial perfusate hemoglobin below (≤5.2 mmol/L) or above (>5.2 mmol/L) the median. The association between hemoglobin levels and EAD or recipient peak transaminase levels was assessed.

**Results:**

Among 23 livers, eight were considered unsuitable for transplantation, leaving 15 livers for assessment. Higher initial hemoglobin was associated with a lower risk of EAD (0% vs. 55.6%, *p* = 0.04). Perfusate hemoglobin decreased after NMP initiation (*p* = 0.003) and negatively correlated with recipient peak transaminase levels (ALT: *ρ* = −0.72, *p* = 0.002; AST: *ρ* = −0.79, *p* < 0.001). Consistently, higher hemoglobin livers also demonstrated lower perfusate liver enzymes.

**Conclusions:**

Perfusate hemoglobin levels decreased during NMP, and lower perfusate hemoglobin levels were associated with a higher incidence of EAD and higher levels of liver injury markers. Maintaining higher hemoglobin levels during NMP may help reduce ischemia–reperfusion injury and prevent or attenuate EAD. Larger prospective studies are needed to validate the findings of this pilot study.

## Graphical abstract

Perfusate hemoglobin levels decrease during normothermic machine perfusion and were associated with postoperative early allograft dysfunction or transaminase levels of recipients. Future studies to validate the findings from this pilot study are warranted.

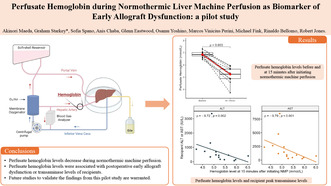

## 1 INTRODUCTION

There is a disparity between organ demand and supply in liver transplantation, resulting in increased waiting list mortality.^1^ This has led to the acceptance of livers from higher risk donations, such as donation after cardiac death (DCD).^2^, ^3^ However, these higher risk organs may be at greater risk of ischemia–reperfusion injury (IRI), clinically manifested as early allograft dysfunction (EAD).^4^, ^5^, ^6^

EAD is the result of the complex interplay between donor, procurement, preservation, and perioperative recipient factors. However, machine perfusion techniques may improve preservation factors by perfusing the donated livers with oxygen and nutrients,^7^, ^8^, ^9^ thereby suppressing IRI.^4^, ^5^, ^6^, ^8^ This approach is becoming more widely available concomitant with the acceptance of livers from higher risk donors.

Among available machine perfusion techniques, normothermic machine perfusion (NMP) may be particularly effective and has been investigated^10^, ^11^ with a particular focus on biomarkers, which, when measured in the perfusate, may help predict posttransplant liver function.^12^, ^13^, ^14^, ^15^ However, clinically, the association of such biomarkers with EAD remains only partly investigated.^16^, ^17^ Among several potential biomarkers, perfusate hemoglobin levels during NMP have the potential for further assessment as they are an easily obtainable and modifiable determinant of oxygen delivery during NMP. In this regard, low perfusate hemoglobin levels may adversely affect liver oxygen delivery and increase the risk of EAD. Yet, limited attention has been paid to perfusate hemoglobin levels.

Accordingly, we hypothesized that lower perfusate hemoglobin levels would be associated with a greater risk of posttransplant EAD and conducted a retrospective analysis utilizing systematically collected laboratory variables to investigate the change in perfusate hemoglobin levels during NMP and its association with posttransplant EAD.

## 2 MATERIALS AND METHODS

### 2.1 Trial design and ethical oversight

This pilot retrospective cohort study was conducted according to the Strengthening the Reporting of Observational Studies in Epidemiology (STROBE) guidelines.^18^ Ethical approval was obtained from the Austin Health Human Research Ethics Committee (HREC/91110/Austin‐2022). Because no additional interventions were undertaken and analysis was performed on anonymized data, the need for informed consent was waived.

### 2.2 Selection of included livers

We included all livers treated with NMP between July 2020 and August 2022 at our tertiary liver transplant center, where an average of 68 adult liver transplantations are performed annually during the years of the study period. The decision to initiate NMP was made based on consensus among the liver transplant surgeons. OrganOx Metra™ (OrganOx limited, Oxford, United Kingdom) device was used for all NMPs.

### 2.3 Organox device setup and perfusion

As we covered a large geographic area, NMP was initiated on procured livers at our center after transportation from the donor's hospital under static cold storage with the University of Wisconsin solution, following the “back‐to‐base” approach.^19^, ^20^, ^21^ Before initiating NMP, the livers underwent portal vein and hepatic artery flush with 1 L of 4% albumin.

The OrganOx Metra device was setup following the manufacturer's instructions. The circuit was primed with 3 units of packed red blood cells and 500 mL of 4% albumin solution. Before graft perfusion, the circuit was warmed to 37°C and the acid–base balance status was titrated by 8.4% sodium bicarbonate infusion to achieve a pH of 7.3. During preparation, cefazolin, gentamicin, calcium gluconate, and heparin were added to the circuit according to the manufacturer's instructions. Sodium taurocholate, epoprostenol, heparin, insulin, sodium bicarbonate, and lipid‐free parenteral nutrition were infused during graft perfusion. Additionally, 8.4% sodium bicarbonate was added as a bolus at the commencement of perfusion to correct pH to >7.1. Lipid‐free parental solution was infused when the glucose level dropped below 10 mol/L.

### 2.4 Data collection

The demographic details of the donor and recipient were collected from the donor offer documentation and the electronic medical record. Throughout NMP, blood gas measurements were performed every hour using a hospital blood gas analyzer (ABL 800FLEX, Radiometer, Copenhagen, Denmark), and comprehensive laboratory tests analyzed at the hospital central pathology were performed every 2 h, starting from 15 min after initiating NMP. Blood samples were drawn from the post‐oxygenator catheter toward the hepatic artery. Routine blood tests were performed in the posttransplant period and all these measurement results were also collected. Hemoglobin and carboxyhemoglobin levels from the blood gas measurements were reported and utilized for analysis.

### 2.5 Definitions

We utilize the criteria defined by Olthoff et al.^22^ to diagnose EAD, defined as the presence of one or more of the following: (1) bilirubin ≥10 mg/dL on posttransplant day 7, (2) INR ≥1.6 on posttransplant day 7, or (3) ALT or AST >2000 IU/L within the first 7 days.

Livers were stratified by the initial perfusate hemoglobin levels at 15 min after initiating NMP. Livers with hemoglobin levels above the median (5.2 mmol/L) were defined as the high hemoglobin livers, while those below the median were defined as the low hemoglobin livers.

Functional warm ischemia time (fWIT) was defined as the time from the onset of hypoxia (oxygen saturation <50%) or hypoperfusion (systolic blood pressure <50 mm Hg) until the start of cold perfusion in DCD donors.^21^ Liver weights were measured preoperatively, or alternatively, calculated from the liver volume obtained from early posttransplant liver imaging performed clinically as needed, using the liver analysis application on the *Philips IntelliSpace*. For this calculation, liver weights were calculated by multiplying the volumes by 0.91 as previously described.^23^ Minutely oxygen delivery was calculated using blood flow data and oxygen partial pressure (pO_2_) measured every minute by the OrganOx machine.

We linearly interpolated hemoglobin/SpO_2_ from hourly blood gas results, following the equation: Oxygen delivery (mL/min) = (Liver artery flow and portal vein flow (dL/min)) × (1.34 × SpO_2_ × Hemoglobin (g/dL) + PO_2_ (mm Hg) × 0.0031).

### 2.6 Outcomes

The primary outcome of this study was the occurrence of EAD in high and low hemoglobin livers.

The secondary outcomes included change in hemoglobin levels from pre‐perfusion to 5 h after initiating NMP, correlation between the peak transaminase levels in the first seven posttransplant days with the perfusate hemoglobin levels at 15 min after initiating NMP, and change in perfusate lactate, AST, ALT, and LDH in high and low hemoglobin livers. Calculated oxygen delivery in high and low hemoglobin livers and the correlation between mean oxygen delivery over the first 5 h and posttransplant peak transaminase levels were also included.

As a sensitivity analysis, we investigated the correlation between the perfusate hemoglobin levels at 15 min after initiating NMP and peak transaminase levels in the first seven posttransplant days, divided by the liver weight in livers with weight data available.

Finally, as an exploratory and hypothesis‐generating outcome, we assessed the change in perfusate carboxyhemoglobin (CO‐Hb) levels as a possible additional mechanistic explanation for changes in hemoglobin during NMP. This analysis was performed because CO‐Hb is a clinically validated and pathophysiologically accepted marker of hemolysis formed by the binding of hemoglobin with carbon monoxide from heme breakdown.^24^, ^25^, ^26^ Moreover, CO‐Hb can be routinely measured from blood gas analysis, allowing serial assessment.

### 2.7 Statistical analysis

Descriptive statistics were used to summarize patient characteristics. Continuous variables were reported as medians with interquartile range (IQR) and categorical variables were reported as proportions. Fisher's exact tests and nonparametric Mann–Whitney *U* tests were performed for categorical and continuous variables, respectively. Spearman's correlation was used to determine the presence of a correlation between variables. We also constructed a linear mixed model adjusted for multiple measurements in each liver to assess hemoglobin level or carboxyhemoglobin level changes over time. A *p* < 0.05 was considered statistically significant. Due to the exploratory nature of the study, no *p*‐value adjustments were applied for multiple comparisons. All statistical analyses were performed using R version 4.2.1.^27^

## 3 RESULTS

### 3.1 Characteristics of donors and liver preservation

A total of 23 procured liver grafts underwent NMP during the study period. Table 1 presents the baseline characteristics of the donors and the preservation. There were three donors over 60 years old (13.0%) with the median donor age of 47 years, and the median donor risk index^28^ was 1.80. DCD was the dominant organ donation pathway (*n* = 18, 78.3%). Donor baseline characteristics were comparable between high and low hemoglobin livers. High hemoglobin livers showed nonsignificantly lower liver weights (1680 g vs. 1988 g, *p* = 0.30).

**TABLE 1:** Donor demographics and preservation characteristics according to initial hemoglobin level above or below the median.

	All livers	High Hb livers^a^	Low Hb livers^a^	*p*‐value
	*N* = 23	*N* = 11	*N* = 12	
Donor demographics				
Donor age (years)^b^	47 [27, 55]	51 [29, 57]	45 [24, 53]	0.54
Male donor^c^	19 (82.6)	7 (63.6)	12 (100.0)	0.04
Donor type				
Donation after brain death^c^	5 (21.7)	3 (27.3)	2 (16.7)	0.64
Donation after cardiac death^c^	18 (78.3)	8 (72.7)	10 (83.3)	
Donor peak ALT (IU/L)^b^	53 [24, 197]	55 [24, 90]	48 [24, 351]	0.67
Donor last ALT (IU/L)^b^	36 [20, 83]	40 [24, 83]	33 [17, 88]	0.64
Donor risk index^b^	1.80 [1.46, 2.02]	1.77 [1.35, 2.12]	1.81 [1.62, 1.99]	0.76
Downtime (min)^b^	0 [0.0, 19.0]	0 [0.0, 8.5]	0 [0.0, 24.3]	0.45
Liver weight (g)^d^	1920 [1605, 2118]	1680 [1415, 2118]	1988 [1760, 2173]	0.30
Preservation				
Time from withdrawal to perfusion (min)^b^, ^e^	26.0 [23.5, 28.0]	28.0 [28.0, 29.0]	24.0 [22.0, 25.0]	<0.001
Functional warm ischemia time (min)^b^, ^e^	22.5 [18.3, 23.8]	23.5 [21.5, 26.0]	21.0 [18.3, 22.8]	0.13
Cold ischemia time (min)^b^	265 [230, 299]	233 [217, 269]	280 [260, 339]	0.03
Perfusion time (min)^b^, ^f^	902 [650, 1020]	650 [559, 735]	997 [902, 1167]	0.045
Bile production^c^	22 (95.7)	10 (90.9)	12 (100.0)	0.48
Hb at 15 min after NMP start (mmol/L)^b^	5.2 [4.8, 5.6]	5.6 [5.4, 5.9]	4.8 [4.5, 5.1]	<0.001
Mean Hb during NMP (mmol/L)^b^, ^g^	4.6 [4.3, 5.1]	5.1 [4.9, 5.5]	4.3 [4.0, 4.5]	0.001
Transplanted^c^	15 (65.2)	6 (54.5)	9 (75.0)	0.40

Abbreviations: ALT, alanine aminotransferase; AST, aspartate aminotransferase; Hb, hemoglobin; NMP, normothermic machine perfusion.

^a^ Livers were stratified by the Hb levels at 15 min after initiating NMP with the cutoff of the median initial Hb of all organs after NMP start (5.2 mmol/L).

^b^ Median and interquartile range are reported.

^c^ Frequency and percentages are reported.

^d^ Liver weights were available in 15 livers (7 high Hb livers and 8 low Hb livers).

^e^ Applied to DCD livers; *n* = 18 (8 high Hb livers, 10 low Hb livers).

^f^ Applied to transplanted livers; *n* = 15 (6 high Hb livers, 9 low Hb livers).

^g^ Mean Hb during the first 5 h of NMP are reported.

Fifteen livers (65.2%), including six high hemoglobin livers and nine low hemoglobin livers, were transplanted following NMP. The reasons for discarded livers are described in Table S1. The median cold ischemia time (CIT) among all perfused livers was 265 min. Among livers from DCD donors, median fWIT and time from withdrawal of support to cold perfusion were 22.5 and 26.0 min. While low hemoglobin livers were exposed to longer CIT and perfusion duration, high hemoglobin livers were exposed to longer time from withdrawal to perfusion. Median hemoglobin levels during NMP were significantly higher (*p* < 0.001) in high hemoglobin livers compared to low hemoglobin livers.

### 3.2 Recipient demographics and post‐transplant outcomes

Table 2 presents the baseline characteristics of recipients and their posttransplant outcomes. The median recipient age was 61 years and the leading cause for transplantation was alcohol‐related liver disease (*n* = 5, 33.3%). Na Model for End‐Stage Liver Disease (MELD‐Na) score was 19 as the median and was higher in recipients with high hemoglobin livers (26 vs. 17, *p* = 0.03). Among 15 recipients, two died before hospital discharge (13.3%) and Table S2 shows detailed information on their hospital courses.

**TABLE 2:** Recipient demographics and posttransplantation observations.

	Transplanted livers	High Hb livers^a^	Low Hb livers^a^	*p*‐value
	*N* = 15	*N* = 6	*N* = 9	
Demographics				
Recipient age (years)^b^	61 [56, 67]	56 [52, 58]	66 [61, 67]	0.045
Male recipient^c^	13 (86.7)	5 (83.3)	8 (88.9)	>0.99
Cause of liver failure^c^				0.50
Alcohol‐related liver disease	5 (33.3)	2 (33.3)	3 (33.3)	
Nonalcoholic steatohepatitis	3 (20.0)	2 (33.3)	1 (11.1)	
Hepatitis C virus	2 (13.3)	0 (0.0)	2 (22.2)	
Alcohol‐related liver disease and Hepatitis C virus	2 (13.3)	0 (0.0)	2 (22.2)	
Primary biliary cholangitis	1 (6.7)	1 (16.7)	0 (0.0)	
Histiocytosis	1 (6.7)	1 (16.7)	0 (0.0)	
Alpha‐1 antitrypsin deficiency	1 (6.7)	0 (0.0)	1 (11.1)	
MELD score^b^	17 [9.5, 23.5]	23 [18, 29]	17 [6, 17]	0.08
Na MELD score^b^	19 [12, 28]	26 [23, 30]	17 [6, 19]	0.03
Posttransplant outcomes				
Peak ALT (IU/L)^b^, ^d^	439 [236, 705]	157 [144, 346]	688 [439, 872]	0.01
Peak AST (IU/L)^b^, ^d^	948 [578, 2390]	462 [338, 687]	2367 [1193, 2649]	0.003
PT‐INR^b^, ^e^	1.0 [1.0, 1.2]	1.0 [1.0, 1.0]	1.0 [0.9, 1.2]	0.76
Total bilirubin (μmol/L)^b^, ^e^	31 [15, 53]	31 [21, 42]	31 [12, 61]	0.95
Early allograft dysfunction^c^	5 (33.3)	0 (0.0)	5 (55.6)	0.04
Alive at hospital discharge^c^	13 (86.7)	5 (83.3)	8 (88.9)	>0.99
Postoperative biliary stenosis^c^, ^f^	6 (46.2)	2 (40.0)	4 (50.0)	>0.99
Alive at 1 year^c^	13 (86.7)	5 (83.3)	8 (88.9)	>0.99

Abbreviations: ALT, alanine aminotransferase; AST, aspartate aminotransferase; Hb, hemoglobin; MELD, model for end‐stage liver disease; PT‐INR, international normalized ratio of prothrombin time.

^a^ Livers were stratified by the Hb levels at 15 min after initiating NMP with the cutoff of the median initial Hb of all organs after NMP start (5.2 mmol/L).

^b^ Median and interquartile range are reported.

^c^ Frequency and percentages are reported.

^d^ Peak values over the first 7 days after transplantation are reported.

^e^ Values at posttransplant day seven are reported.

^f^ Applied to livers of alive patients at hospital discharge; *n* = 13 (5 high Hb livers, 8 low Hb livers).

Regarding posttransplant outcomes, the recipients' peak ALT and AST over the first 7 days after transplantation were lower in recipients with high hemoglobin livers compared to recipients with low hemoglobin livers (ALT: 157 IU/L vs. 688 IU/L, *p* = 0.01; AST: 462 IU/L vs. 2367 IU/L, *p* = 0.003). No significant difference was observed regarding long‐term patient outcomes between recipients with High and low hemoglobin livers. However, EAD, the primary outcome of this study, did not occur in the six recipients with high hemoglobin livers and, in contrast, it occurred in five out of nine recipients with low hemoglobin livers (*p* = 0.04). Consistently, low hemoglobin livers showed numerically worse Model for Early Allograft Function Scoring (MEAF) score compared to high hemoglobin livers (median MEAF score: 5.33 vs. 3.32, *p* = 0.41) as shown in Figure S1.

### 3.3 Change in hemoglobin levels from pre‐perfusion to 5 h after initiating NMP

**FIGURE 1:**
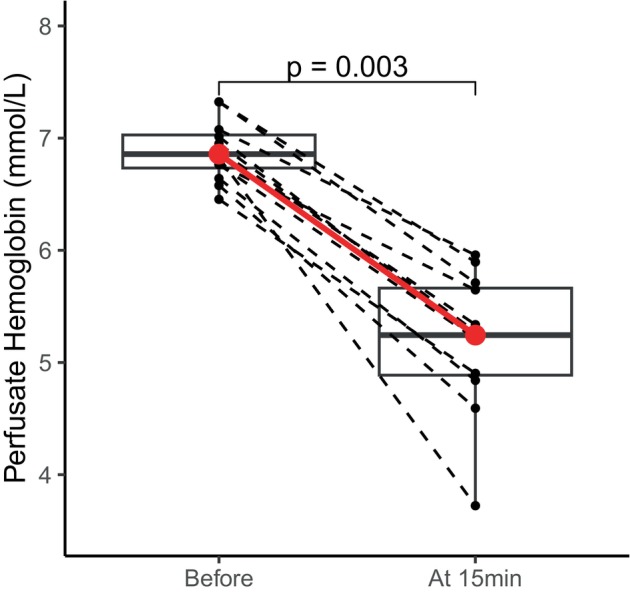
Perfusate hemoglobin levels before and at 15 min after initiating normothermic machine perfusion. Each dashed line represents an individual liver and a significant decrease in median hemoglobin levels was observed in 12 livers with available pre‐perfusion hemoglobin data (red line, *p* = 0.003, Mann–Whitney *U* test). [Color figure can be viewed at wileyonlinelibrary.com]

**FIGURE 2:**
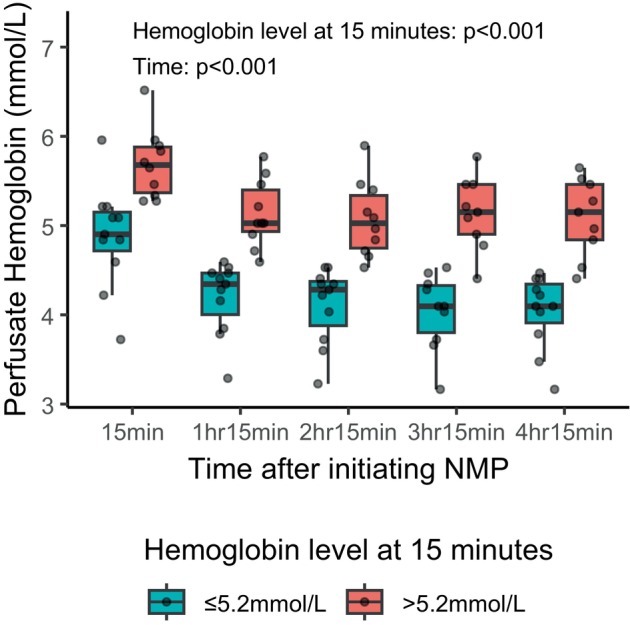
Perfusate hemoglobin levels according to initial hemoglobin level after initiating normothermic machine perfusion. Longitudinal analysis of perfusate hemoglobin levels during the first 5 h of NMP in all 23 livers. A significant decrease in hemoglobin levels over time was observed (*p* < 0.001), and livers with initial hemoglobin levels above the median exhibited significantly higher hemoglobin levels, with a mean difference of 0.9 mmol/L over time (*p* < 0.001, linear mixed model). NMP, Normothermic machine perfusion. [Color figure can be viewed at wileyonlinelibrary.com]

Pre‐perfusion perfusate hemoglobin levels were available for 12 livers. Among these livers, perfusate hemoglobin levels significantly decreased after initiating liver perfusion (from 6.9 mmol/L to 5.2 mmol/L, *p* = 0.003) as shown in Figure 1. Moreover, linear mixed modeling showed that those livers with initial hemoglobin levels above the median had significantly higher hemoglobin levels throughout NMP, with a difference of 0.9 mmol/L over time (*p* < 0.001) as shown in Figure 2. We did not find a significant correlation between initial hemoglobin levels and liver weights as shown in Figure S2 (*ρ* = −0.3, *p* = 0.28).

### 3.4 Association of perfusate hemoglobin levels with post‐transplant recipient transaminases

**FIGURE 3:**
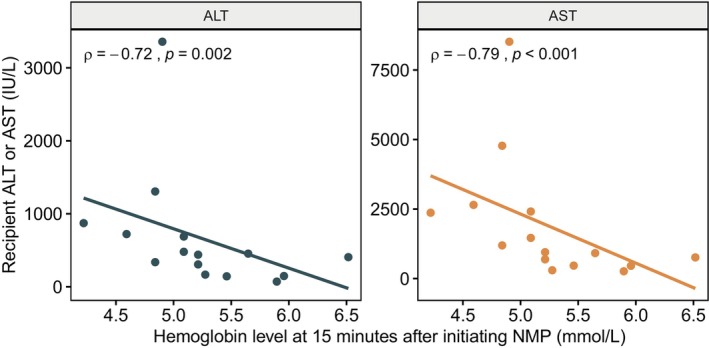
Spearman's correlation between initial hemoglobin after initiating normothermic machine perfusion and recipient peak transaminase levels. In 15 transplanted livers, perfusate hemoglobin levels at 15 min after initiating NMP were negatively correlated with posttransplant recipient transaminase values (black dot and line with ALT: *ρ* = −0.72, *p* = 0.003; gold dot and line with AST: *ρ* = −0.79, *p* < 0.001, Spearman's correlation). ALT, alanine aminotransferase; AST, aspartate aminotransferase; NMP, normothermic machine perfusion. [Color figure can be viewed at wileyonlinelibrary.com]

In the 15 transplanted livers, perfusate hemoglobin levels at 15 min after initiation of NMP were negatively correlated with peak posttransplant recipient transaminase values (with ALT: *ρ* = −0.72, *p* = 0.003; with AST: *ρ* = −0.79, *p* < 0.001) as shown in Figure 3. This negative correlation was even stronger when transaminase levels were divided by liver weight in 13 transplanted livers with available data (Figure S3). Other correlations between perfusate or biliary variables during NMP and posttransplant transaminase values were also found (Table S3). However, at 15 min, they were all less strongly associated with outcomes than perfusate hemoglobin. Throughout the first 5 h of NMP, only glucose showed an equivalent correlation with posttransplant transaminase values, while lactate's correlation was low, and that of ALT and AST, although present, was inferior to that of hemoglobin.

### 3.5 Association of perfusate hemoglobin levels with perfusate liver enzymes

**FIGURE 4:**
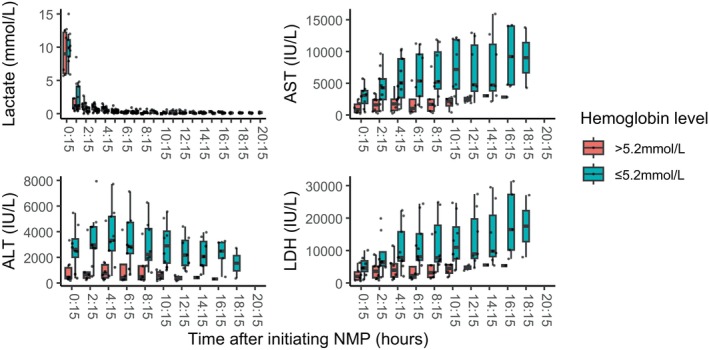
Perfusate biochemistry over time in transplanted livers during NMP. These boxplots described the change in biochemistries over time according to the initial hemoglobin levels (Red: Initial hemoglobin level >5.2 mmol/L, Blue: Initial hemoglobin level <5.2 mmol/L) in transplanted livers. ALT, alanine aminotransferase; AST, aspartate aminotransferase; LDH, lactate dehydrogenase; NMP, normothermic machine perfusion. [Color figure can be viewed at wileyonlinelibrary.com]

Figure 4 and Figure S4 show the results of serial biochemical tests of the perfusate during the whole duration of NMP for the transplanted livers and all perfused livers. Perfusate ALT, AST, and lactate dehydrogenase (LDH) values increased during NMP in both groups, and the median values of these parameters consistently favored High hemoglobin livers.

### 3.6 Oxygen delivery and its association with posttransplant recipient transaminases

Figure S5 describes calculated oxygen delivery in 15 transplanted livers. Oxygen delivery was higher in high hemoglobin livers (*p* = 0.04). Oxygen delivery was also negatively correlated with peak posttransplant recipient transaminase values (with ALT: *ρ* = −0.69, *p* = 0.006; with AST: *ρ* = −0.72, *p* = 0.004) as shown in Figure S6.

### 3.7 Perfusate carboxyhemoglobin levels and its association with perfusate hemoglobin levels

**FIGURE 5:**
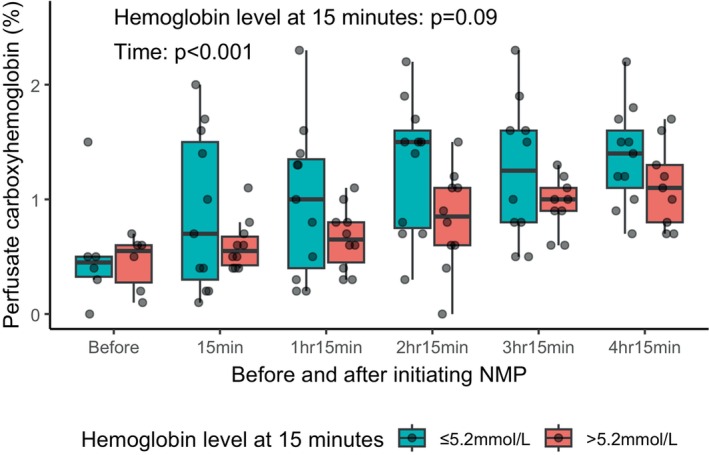
Perfusate carboxyhemoglobin level over time according to initial hemoglobin level after initiating normothermic machine perfusion. The perfusate carboxyhemoglobin level, obtained in 21 livers, demonstrates an increase over time (*p* < 0.001). Additionally, the median carboxyhemoglobin levels remain consistently lower in livers with an initial hemoglobin level above the median, with a mean difference of 0.3% (*p* = 0.09, linear mixed model). ALT, alanine aminotransferase; AST, aspartate aminotransferase; LDH, lactate dehydrogenase; NMP, normothermic machine perfusion. [Color figure can be viewed at wileyonlinelibrary.com]

Finally, in 21 livers with available data, the perfusate CO‐Hb significantly increased over time during NMP as shown in Figure 5 (linear mixed model, *p* < 0.001). CO‐Hb levels were numerically lower in high hemoglobin livers compared with low hemoglobin livers (*p* = 0.09).

## 4 DISCUSSION

### 4.1 Key findings

To the best of our knowledge, this pilot study is the first to report that livers with higher perfusate hemoglobin levels were less likely to develop EAD. Perfusate hemoglobin levels decreased shortly after initiating NMP and remained stable afterward. In addition, perfusate hemoglobin levels were strongly and negatively correlated with posttransplant transaminase levels and these associations were robust to adjustment for liver weight. Consistently, livers with higher hemoglobin also demonstrated lower perfusate liver enzyme levels. Moreover, perfusate oxygen delivery was also negatively correlated with posttransplant transaminase levels. Finally, as an exploratory outcome, carboxyhemoglobin increased during NMP suggesting increased heme release.

### 4.2 Relationship with previous studies

NMP has been developed to suppress IRI by minimizing cold storage and perfusing livers with oxygen.^4^, ^5^, ^6^, ^8^ As IRI clinically manifests as EAD,^4^, ^5^, ^6^ it is important to identify perfusate biomarkers for livers at risk of EAD and/or therapeutic targets during perfusion to help mitigate EAD. However, to the best of our knowledge, only two other studies have attempted to identify perfusate variables during NMP, which might show an association with early dysfunction of the transplanted livers.^16^, ^17^ Our findings on other perfusate variables broadly aligned with these studies. However, we are the first to focus on the association between hemoglobin and EAD or posttransplant transaminase levels. The increasing acceptance of higher risk livers^2^, ^29^ also highlights the growing importance of investigations on EAD or IRI. Livers from elderly donors and DCD donors have been reported to be more vulnerable to IRI,^4^, ^5^, ^6^ and over 80% of livers in this study fell into these higher risk categories. We employed a “back‐to‐base” approach at our center, necessitating some degree of cold ischemia time for all livers.^19^, ^20^, ^21^ This additional vulnerability to IRI from cold storage further enabled our ability to identify perfusate variables associated with the development of EAD.

Our findings are theoretically sound as the primary purpose of employing NMP is to reduce the IRI by perfusing the organ with oxygenated blood,^4^, ^5^, ^6^, ^8^, ^30^ and, accordingly, we demonstrated that livers with higher perfusate hemoglobin received higher oxygen delivery. However, Watson et al. reported that NMP at partial pressure of oxygen (pO_2_) >600 mm Hg was associated with a higher incidence of blood pressure fall after the reperfusion in the recipients, compared to NMP at pO_2_ of 150–200 mm Hg.^31^ Their findings imply that high pO_2_, another component of oxygen delivery, may exacerbate the IRI. Thus, the Orgnox metra device standardly sets pO_2_ within 90–150 mm Hg,^31^ which is well below the ranges associated with possible toxicity in their study. Our findings suggest that higher hemoglobin levels likely contribute to higher oxygen delivery under the same pO_2_ and blood flow. As such, among the components of oxygen delivery, investigating optimal hemoglobin levels during NMP to prevent further liver injury is warranted.

There are preclinical studies that investigated optimal hemoglobin levels during NMP. A porcine study^32^ reported that a lower hemoglobin concentration was associated with lower oxygen delivery, lower oxygen consumption, and higher transaminase levels during NMP. They concluded that hemoglobin levels of at least 1.24 or 1.86 mmol/L should be maintained for liver metabolism. Another porcine study in kidney NMP^33^ reported that hemoglobin levels of only up to 6 mmol/L improved graft function. Our study findings with the threshold of 5.2 mmol/L broadly align with these preclinical findings. However, our study is the first investigation in human NMP, showing the association of perfusate hemoglobin levels with liver injury during perfusion and the posttransplant period.

Regarding the decrease in perfusate hemoglobin, only one previous study reported initial hematocrit levels after NMP initiation.^12^ In this study, hemoglobin levels estimated from measured hematocrit levels were 5–6 mmol/L, similar to the levels observed in our study. However, the occurrence of EAD was not evaluated as livers were not transplanted. Dilution of the perfusate with the remaining flush fluid is expected to be a main reason for this decrease, supported by numerically higher liver weights in low hemoglobin livers. In addition, extravasation or trapping of red cells,^34^ hemolysis^35^ suggested by increased LDH and CO‐Hb, or a combination of these potential causes may also contribute to the observed hemoglobin decrease. Further investigation of the underlying mechanisms for this hemoglobin decrease, potentially with histological evaluation or measurements of other relevant parameters, will help understand whether perfusate hemoglobin is a marker of liver injury, in which case maintaining higher levels may not be beneficial, or whether it also represents a potential therapeutic target.

Finally, we observed CO‐Hb increase during NMP. CO‐Hb is a marker for hemolysis and has also been proposed as a potential marker of IRI through the upregulation of hemeoxygenase‐1 during IRI.^36^ However, perfusate CO‐Hb levels are also inevitably affected by CO‐Hb levels in the packed red blood cells^37^ used for circuit preparation. The interplay between hemolysis, IRI, and residual CO‐Hb from the blood product introduces complexity, so our findings can only serve as hypothesis‐generating. Additional research is needed to disentangle these factors and better characterize the relationship between CO‐Hb dynamics and hepatocellular injury during NMP.

### 4.3 Implications of study findings

Our findings suggest that high perfusate hemoglobin levels are a potential biomarker of increased risk of EAD during NMP. Moreover, as hemoglobin levels are modifiable, our findings suggest the potential for controlled trials to test whether targeting higher hemoglobin levels during NMP can decrease the incidence of EAD. Finally, as the hemoglobin levels reliably fall within 15 min of initiating NMP, our observations suggest the need for such hemoglobin adjustments to be considered early in the course of NMP.

### 4.4 Study strengths and limitations

Our study has several strengths. First, we analyzed granular and detailed longitudinal data using the well‐established and widely studied OrganOx Metra system for liver perfusion,^9^, ^19^, ^38^ increasing the applicability of our findings. Second, our findings on other biochemical variables are aligned with previous research investigating various biochemical variables as predictors of liver injury at early posttransplant stage,^16^, ^17^, ^39^ providing a degree of external validity. Third, perfusate hemoglobin levels demonstrated a consistent negative correlation with the ratio of transaminase levels to liver weight. This likely reflects the degree or intensity of liver injury per unit of liver tissue, while transaminase levels themselves likely reflect overall liver injury. The consistent result in this sensitivity analysis may be more relevant and supports our main findings. Finally, these comparative analyses of perfusion variables in relation to liver injury during and at early stage after machine perfusion address an important knowledge gap in the literature. Our findings provide new insights into the role of perfusate hemoglobin levels during NMP, further supported by the difference in oxygen delivery according to perfusate hemoglobin levels and by the negative correlation between oxygen delivery and posttransplant peak transaminase levels.

However, we acknowledge some limitations. First, this pilot retrospective study has a small sample size, which increases the risk of type I and type II error and is underpowered to investigate associations with long‐term outcomes. Also, this is a single‐center study following a specific protocol. Therefore, the generalizability of our findings is untested. However, our study demonstrated strong and significant correlations of perfusate hemoglobin or oxygen delivery with posttransplant peak transaminase levels and EAD occurrence. Second, the baseline differences in CIT, perfusion duration, and Na‐MELD score can affect our findings. Thus, together with the nature of the study, we acknowledge that our study is only hypothesis‐generating. Third, we selected EAD as per Olthoff's criteria as our primary outcome because it incorporates transaminase levels as a key component to reflect the extent of IRI, the main focus of our study. Though EAD as per Olthoff or elevated posttransplant transaminase levels showed associations with long‐term graft outcomes,^22^, ^40^ other scoring systems may carry stronger predictive ability for long‐term graft survival. In this regard, low hemoglobin livers also showed numerically worse MEAF scores, supporting our main findings. Fourth, a comprehensive assessment of IRI, including mitochondrial function or oxygen consumption, was not performed, and information on histological assessments of grafts, viscosity measurement of the perfusate fluid, and shelf‐life of the packed red blood cells was not collected. Such information needs to be assessed in future trials to more deeply understand the role of perfusate hemoglobin and oxygen delivery in suppressing IRI or EAD.

## 5 CONCLUSION

In this pilot study, we found that perfusate hemoglobin levels decreased significantly after initiating NMP and that lower hemoglobin levels were associated with posttransplant EAD. This association was stronger than that seen for other widely used perfusate biomarkers. These results indicate perfusate hemoglobin level may be a risk factor or biomarker for EAD in this setting. They also imply the need for further research to validate these observations in larger cohorts. Finally, our findings justify trials aimed at exploring the potential value of maintaining higher hemoglobin levels during NMP.

## AUTHOR CONTRIBUTIONS

Concept/design: All authors. Data collection/management: Akinori Maeda, Graham Starkey, Sofia Spano, Marcos Vinicius Perini, Michael Fink, and Robert Jones. Data analysis/interpretation: Akinori Maeda, Anis Chaba, Graham Starkey, Glenn Eastwood, and Rinaldo Bellomo. Drafting article: Akinori Maeda, Graham Starkey, Osamu Yoshino, and Rinaldo Bellomo. Critical revision of paper: Graham Starkey, Glenn Eastwood, Marcos Vinicius Perini, Michael Fink, Rinaldo Bellomo, and Robert Jones. Final approval of paper: All authors.

## FUNDING INFORMATION

None.

## CONFLICT OF INTEREST STATEMENT

The authors declare they have no conflict of interest in relation to this manuscript.

## Supporting information


Data S1.

## Data Availability

The data that support the findings of this study are available from the corresponding author upon reasonable request.
