# Supplementary material for: Perfusate hemoglobin during normothermic liver machine perfusion as biomarker of early allograft dysfunction: A pilot study
Source: Artif Organs. 2024 Sep 18;49(1):108–18. doi: 10.1111/aor.14862 (PMC11687207; doi:10.1111/aor.14862)
Supplement: Supplementary file 1 — Data S1. [file AOR-49-108-s001.docx]

***Supplementary Materials***

**Perfusate Hemoglobin during Normothermic Machine Perfusion as Biomarker of Early Allograft Dysfunction**

Akinori Maeda, Graham Starkey, Sofia Spano, Anis Chaba, Glenn Eastwood, Osamu Yoshino, Marcos Vinicius Perini, Michael Fink, Rinaldo Bellomo, Robert Jones.

**Table S1. Reasons for discarded livers** **2**

**Table S2. Detailed information on the hospital courses of two deceased recipients3**

**Table S3. Spearman’s correlation coefficient between perfusate or biliary variables during normothermic machine perfusion and post-transplant transaminase in 15 transplanted organs5**

**Figure S1. MEAF score in Low and High hemoglobin livers. 6**

**Figure S2. Spearman’s correlation between initial hemoglobin levels and liver weights. 7**

**Figure S3. Spearman’s correlation between initial hemoglobin after normothermic machine perfusion start and peak recipient transaminase levels divided by liver weight. 8**

**Figure S4. Perfusate biochemistries over time in all perfused livers during NMP. 9**

**Figure S5. Oxygen delivery over time in transplanted livers according to the initial hemoglobin level after initiating normothermic machine perfusion. 10**

**Figure S6**. **Spearman’s correlation between oxygen delivery and post-transplant peak transaminase levels. 11**

**Table S1. Reasons for discarded livers**

|  |  | Discarded livers | | | | | | | |
| --- | --- | --- | --- | --- | --- | --- | --- | --- | --- |
|  |  | 1 | 2 | 3 | 4 | 5 | 6 | 7 | 8 |
| Perfusate findings | High ALT (>5000 ~ >20000) | O | O |  |  |  |  | O | O |
|  | Poor pH correction |  | O |  | O |  |  | O |  |
|  | Poor glucose consumption |  |  |  |  | O |  |  |  |
|  | Poor lactate clearance |  |  |  |  |  |  | O |  |
| Bile findings | No bile production |  |  | O |  |  |  |  |  |
|  | High glucose | O |  |  |  | O |  |  | O |
| Macroscopy findings | Macrosteatosis |  |  |  | O |  | O | O |  |

Livers were usually discarded based on multiple reasons.

ALT, alanine aminotransferase

**Table S2. Detailed information on the hospital courses of two deceased recipients**

|  | **Recipient 1** | **Recipient 2** |
| --- | --- | --- |
| *Demographics* |  |  |
| Age | 49 | 48 |
| Sex | Male | Male |
| Days from hospital admission to transplantation | 1 | 82 |
| Days from transplantation to death | 13 | 30 |
| Diagnosis | Histiocytosis | Alcohol related liver disease / Hepatocellular carcinoma |
| MELD at transplantation | 13 | 17 |
| Na MELD at transplantation | 24 | 19 |
| *Post-transplant parameters* |  |  |
| Peak ALT^a^ | 166 | 3358 |
| Peal AST^a^ | 298 | 8518 |
| PT-INR^b^ | 1 | 1.2 |
| Bilirubin^b^ | 29 | 61 |
| Early allograft dysfunction | No | Yes |
| *Cause of death* | Uncontrollable bleeding during Inferior Vena Cava thrombectomy | Multi-organ failure, gram negative bacteremia in the context of ischemic liver |
| *Hospital course* | The transplanted liver functioned satisfactory; however, the patient developed an occlusive inferior vena cava (IVC) thrombosis following liver transplantation. Despite continuing therapeutic anticoagulation, the thrombosis remained unresolved, necessitating the patient's return to the operating theater for IVC thrombectomy. Unfortunately, during the procedure, significant bleeding ensued, leading to a deterioration in the patient's hemodynamic status. Despite all efforts to control the bleeding, the patient experienced cardiac arrest on the operating table. | The patient was referred to our hospital for continued management of decompensated liver disease. At the time of listing the patient for liver transplantation, he was in a critically severe condition, having previously undergone multiple ICU admissions and experiencing an episode of pulseless electrical activity (PEA) arrest. Despite successful completion of the transplantation procedure, he experienced a significant graft dysfunction with large areas of necrosis and that contributed to his death. |

ALT, alanine aminotransferase; AST, aspartate aminotransferase; MELD, model for end stage liver disease; PT-INR, international normalized ratio of prothrombin time

^a^Peak values over first 7 days after transplantation are reported.

^b^Values at posttransplant day 7 are reported.

**Table S3. Spearman’s correlation coefficient between perfusate or biliary variables during normothermic machine perfusion and post-transplant transaminase in 15 transplanted organs**

ALT, alanine aminotransferase; AST, aspartate aminotransferase; LDH, lactate dehydrogenase; GGT, gamma-glutamyl transferase

*Bile GGT was measured in 14 patients.

***
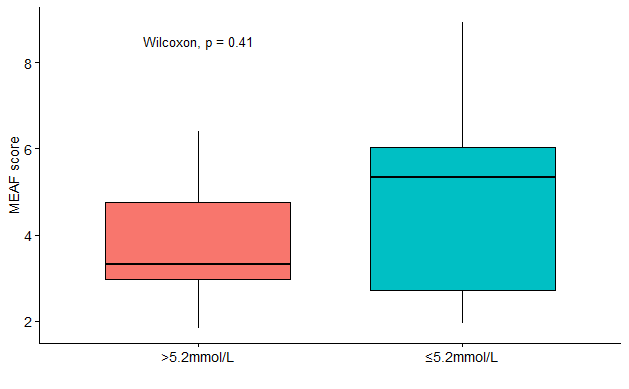
***

**Figure S1. MEAF score in Low and High hemoglobin livers**

Abbreviations: MEAF, Model for Early Allograft Function Scoring

Figure legends: Low Hemoglobin livers showed numerically worse MEAF score compared to High Hemoglobin livers (median MEAF score: 5.33 vs 3.32, p=0.41).

***
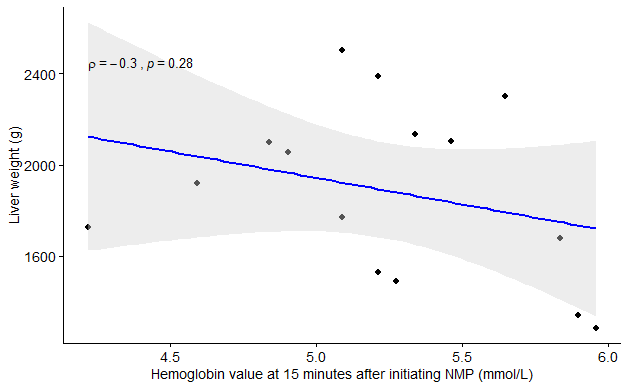
***

**Figure S2. Spearman’s correlation between initial hemoglobin levels and liver weights.**

Abbreviations: NMP, Normothermic machine perfusion

Figure legends: This scatterplot describes the association between the initial hemoglobin levels 15 minutes after the initiation of NMP and liver weights in 15 livers with available information on liver weights. We didn’t find a significant correlation between these parameters (Rho = -0.3, p = 0.28, Spearman’s correlation)

***
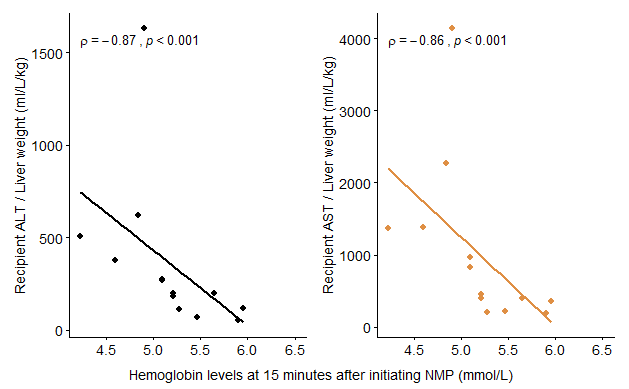
***

**Figure S3. Spearman’s correlation between initial hemoglobin after initiating normothermic machine perfusion and peak recipient transaminase levels divided by liver weight.**

Abbreviations: ALT, alanine aminotransferase; AST, aspartate aminotransferase; NMP, Normothermic machine perfusion

Figure legends: In 13 transplanted livers with available information on liver weight, perfusate hemoglobin levels at 15 minutes after initiating NMP were negatively correlated with peak post-transplant recipient transaminase levels divided by liver weight (Spearman’s correlation).

**
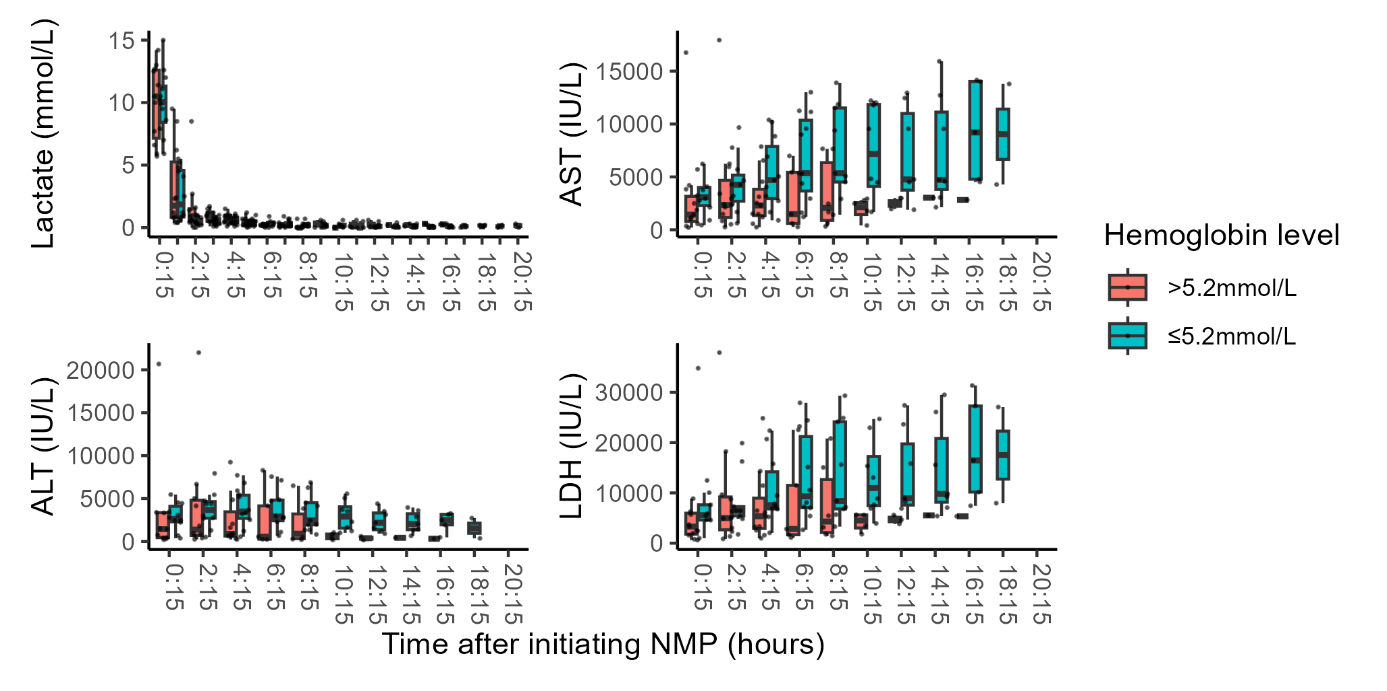
**

**Figure S4. Perfusate biochemistries over time in all perfused livers during NMP.**

Abbreviations: ALT, alanine aminotransferase; AST, aspartate aminotransferase; LDH, lactate dehydrogenase; NMP, Normothermic machine perfusion

Figure legends: These boxplots described the change in biochemistries over time according to the initial hemoglobin levels (Red: initial hemoglobin level >5.2mmol/L, Blue: initial hemoglobin level ≤5.2mmol/L) in all perfused livers. These boxplots showed a similar trend which favored higher perfusate hemoglobin concentrations in transplanted livers (Figure 3).


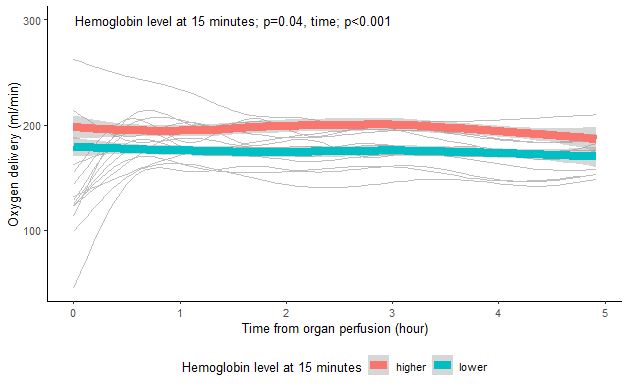


**Figure S5. Oxygen delivery over time in transplanted livers according to the initial hemoglobin level after initiating normothermic machine perfusion**

Abbreviations: NMP, normothermic machine perfusion

Figure legends: Temporal changes in oxygen delivery, smoothed using locally weighted smoothing, are presented for 15 transplanted livers. The mean oxygen delivery was separated over the first five hours of NMP according to the hemoglobin levels at 15 minutes after NMP initiation (represented by blue and red lines). Light gray lines depict the oxygen delivery changes in each liver. Livers with initial hemoglobin levels above the median displayed higher median oxygen delivery compared to those with initial hemoglobin levels below the median over time (p=0.04, linear mixed model).


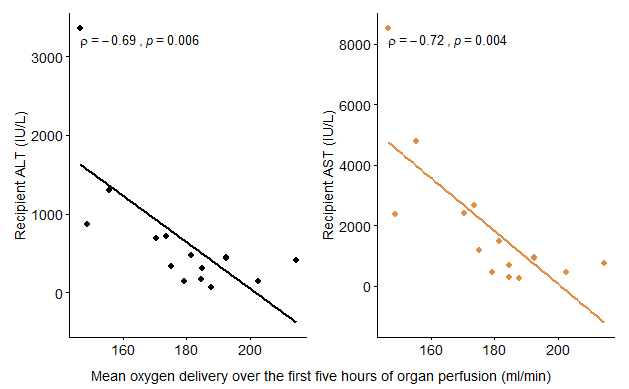


**Figure S6**. **Spearman’s correlation between oxygen delivery and post-transplant peak transaminase levels.**

Abbreviations: ALT, alanine aminotransferase; AST, aspartate aminotransferase

Figure legends: The mean oxygen delivery over the first five hours of perfusion showed a strong negative correlation with post-transplant transaminase values in 15 transplanted livers (with ALT: Rho=-0.69, p=0.006; with AST: Rho=-0.72, p=0.004, Spearman’s correlation).
